# An acceptance divergence? Media, citizens and policy perspectives on autonomous cars in the European Union

**DOI:** 10.1016/j.tra.2022.02.013

**Published:** 2022-04

**Authors:** Fabio Luis Marques dos Santos, Amandine Duboz, Monica Grosso, María Alonso Raposo, Jette Krause, Andromachi Mourtzouchou, Alexandra Balahur, Biagio Ciuffo

**Affiliations:** a European Commission, Joint Research Centre (JRC), Ispra, Italy; b Department of Transportation and Projects and Processes Technology - University of Cantabria, Cantabria, Spain

**Keywords:** Autonomous car, Acceptance, Media analysis, Future mobility

## Abstract

Do citizens, media and policymakers share the same view on autonomous cars? In the present paper, we analyse data from media articles, a Eurobarometer survey, and policy documents, to understand the perspective of different stakeholders when it comes to autonomous cars. We find significant differences between the groups, with a predominance of negative sentiments in news articles and a majority of citizens being wary of autonomous cars, while the political narrative mostly carries a positive tone. The findings highlight a dichotomous perspective about this potentially disruptive technology. This may represent a problem as the benefits of adopting autonomous cars will only come to surface if all actors are engaged and see the advantages they can bring to people’s daily lives. We conclude by encouraging policymakers to promote initiatives to engage citizens in the transformation of road transport and other stakeholders to be advertised the positive implications of autonomous vehicles.

## Highlights

• There is a divergence on connected and automated vehicles views of European citizens and media when compared to policymakers.
• Negative sentiments are prevalent in the media, with most citizens wary of automated vehicles.
• The political narrative mostly carries a positive tone.
• All actors should be engaged to be able to achieve the benefits of adopting automated vehicles.
• Citizen engagement initiatives are encouraged as a means of involving stakeholders in the policymaking process.

## 1 Introduction

Mobility is one of the main pillars of modern society. The possibility, for people and goods, to quickly reach almost any place in the world has fuelled globalisation and unprecedented economic growth in the 19th century. Transport has had wider effects beyond providing seamless and effective mobility: the advent of the car has given birth to automotive cities, transformed public space and has become symbolic of social status. At the same time, the sector generates significant negative externalities for our society. Road accidents, pollutant and greenhouse gas emissions, and productivity losses due to delays and congestion are just the most well-known among them (Alonso Raposo et al., 2019). Attempts to address negative transport impacts usually advocate innovative technological solutions. A clear example regards electric vehicles (EVs), which have been in the market for the last 15 years, but only in 2020 they have started to gain significant market share due to increased driving range they are able to guarantee and the wider availability of recharging points in our cities. These two elements play a key role as they address a major citizens’ concern, the range anxiety (Gómez Vilchez et al., 2019), which considerably reduces the appeal of the “private car” and the perception of freedom and ubiquitous availability attached to it.

In the last decade a new technology has gained attention as a possible tool to achieve the political objective of making transport more efficient and more sustainable: connected and automated vehicles. As enabler of the broader concept of connected, cooperative and automated mobility, they are indeed expected to contribute to make transport supply significantly more efficient and therefore address the ever-increasing mobility demand while reducing transport externalities. However, citizens’ expectations and concerns about this new technology have been only marginally investigated and therefore it is not yet clear how they will react when the new technology will be placed in the market.

Research and innovation (R&I) on these new transport solutions needs to ensure that their implementation is ethically and socially acceptable by having societal actors (e.g. citizens, policymakers and academia) working together to better align R&I processes and outcomes with the values, needs and expectations of society (European Commission, 2018c). According to some recent research, the views of some stakeholders like city officials and citizens, appear to be under-represented in debates on autonomous cars (Van Wynsberghe et al., 2021).

The aim of this article is to analyse the main existing EU policy narratives in the context of autonomous cars and compare them with media and citizens’ opinions. In order to understand the opinions of these two stakeholders (media and citizens), we carry out an analysis of text-based European news media articles (based on sentiment analysis and clustering) and we assess results from a Europe-wide survey by identifying profiles according to different attitudes using the latent class analysis methodology.

### 1.1 Policy background

In Europe, autonomous cars are in the spotlight for policymakers and are proposed as a means to make transport more efficient and to tackle transport safety and emission issues. In the recent European Green Deal (European Commission, 2019) which defines the strategic priorities of the European Commission in the period 2019–2024, clear emphasis is put on the increasing role of automated and connected mobility, together with smart traffic management systems, to contribute to a smart and sustainable mobility. This is not the first example in which automated mobility is included in the European political strategy to make transport more efficient. In the 2018 European Commission communication “On the road to autonomous mobility” (European Commission, 2018a), the vision of autonomous mobility is presented with the ambition “to make Europe a world leader in the deployment of connected and autonomous mobility, making a step-change in Europe in bringing down the number of road fatalities, reducing harmful emissions from transport and reducing congestion (p. 2)”. The communication also states that “even though automated vehicles do not necessarily need to be connected and connected vehicles do not require automation, it is expected that in the medium-term connectivity will be a major enabler for driverless vehicles. Therefore, the Commission will follow an integrated approach between automation and connectivity in vehicles (p. 4)”. In its 2018 Automated Mobility Strategy, which was published as part of the mobility package in May 2018, the European Commission sets the policy framework for the take-up of automated mobility (European Commission, 2018b), and states that it will keep providing financial support to stimulate private investment in the development of technologies and infrastructure linked to autonomous mobility. Lately, in the 2020 Sustainable and Smart Mobility Strategy (European Commission, 2020c), the European Commission sets out a milestone for 2030 to achieve large scale deployment of automated mobility. The strategy puts the focus on a number of key areas for action, including making connected and automated multimodal mobility a reality. There is an overall positive message coming from policy documents and frameworks.

In line with its strategic vision, Europe has considerably invested to support R&I around AVs. Under the Horizon 2020 framework program, for example, AV-related projects are the fourth most funded transport-related projects (Marques dos Santos et al., 2021). Investments will further increase in the new framework programme, Horizon Europe, in which there is an entire group of calls dedicated to connected, cooperative and autonomous mobility and for which a dedicated private-public partnership has been established to support the preparation of the strategic R&I agenda that should support aligning the research to the policy objectives of the European Commission in the years to come (EUCAR, 2021). Furthermore, the European Commission and European Union (EU) Members States (MSs) are putting substantial efforts to lead and contribute to the work of several technical working groups at United Nations Economic Commission for Europe (UNECE) level. The aim is to define global technical regulations that can simplify and foster the market introduction of this new type of technology^1^. The efforts have resulted in the adoption of the first global technical regulation on connected and automated vehicles for motorway applications (United Nations, 2021) which will see its first amendment to enlarge its scope already in 2021.

Beyond their direct contribution in the definition of international technical requirements for AVs, European MSs have also been very active in the last decade to develop national legislations to support real-world testing of AVs. Including Norway and United Kingdom, 6 European Union and associated countries are among the 10 most ready countries for AVs (KPMG, no date). Furthermore, in 2021, both Germany and France have introduced the first framework legislations to allow the deployment of automated shuttles and robo-taxis in urban roads and the EU is working on introducing a regulation during 2022.

All these political and technical activities are promoted in the quest to allow the deployment of AVs in European roads as soon as possible. Their effects will be visible in the upcoming years if people will decide to trust this new technology.

### 1.2 Autonomous vehicle acceptance

While policymakers stress the benefits and advantages of a connected and automated mobility, the actual benefits that AVs can provide, as well as their acceptance by the general public, are debatable.

Studies investigating public opinions towards the willingness to use AVs showed that the acceptance for automated technologies decline as the level of automation increases and that people are not necessarily willing to use fully automated vehicles (Schoettle and Sivak, 2014, Menon et al., 2016, Abraham et al., 2017, Westenberg et al., 2018, Asmussen et al., 2020). In line with this, citizens are in general not willing to purchase AVs, and the technological cost of the technology would need to significantly decrease to change their minds (Bansal and Kockelman, 2017, Talebian and Mishra, 2018).

Surveys from the American Automobile Association (Edmonds, 2018, Edmonds, 2019) demonstrated that the negative attitudes towards the use of AVs is slowly decreasing over time. At the same time, although some studies have shown that a low level of acceptance can constitute the main barrier to the adoption of AVs (Noy et al., 2018, Xu et al., 2018, Liu et al., 2019), others found that people may wait before using the technology, identifying themselves as late adopters (Zmud and Sener, 2017, Panagiotopoulos and Dimitrakopoulos, 2018). This comes together with field research that show the positive impact of the use of shuttles on the willingness to use AVs, pointing out the impact of experience on the acceptance (Piao, et al., 2016, Bernhard et al., 2020, Shi et al., 2021).

Understanding the factors that lead to acceptance of automated cars by the general public is not only necessary, but of great importance to a successful deployment of this new technology. Different aspects influencing people’s acceptance towards AVs, both negatively and positively, have been highlighted in different studies (Bornholt and Heidt, 2020). Among the factors influencing acceptance, they have pointed out socio-demographic characteristics: those more willing to adopt AVs are men, young, have a higher level of education and income, and live in large household (Hohenberger et al., 2016, Abraham et al., 2017, Dong et al., 2019, Hudson et al., 2019). From the side of psychosocial factors, it was found that the perceived ease of use, the perceived usefulness, the perceived safety risk and the initial trust towards the technology are good predictors of the intention to use AVs (Madigan et al., 2017, Buckley et al., 2018, Molnar et al., 2018, Zhang et al., 2019, Zhang et al., 2020). Finally, public opinion surveys found that people are concerned about safety (Menon et al., 2016, Zmud and Sener, 2017), with safety being an important aspect in critical manoeuvres such as lane changing (Ali et al., 2021), which decreases their willingness to use AVs (Dong et al., 2019).

## 2 Methods

### 2.1 Sentiment analysis of news media

The objective of this analysis is to understand the view of text-based news media with relation to AVs, separating the news articles in categories and sentiments. For this purpose, the procedure to identify relevant news articles will be explained first, followed by a description of the model used to determine the views of the articles, which is based on sentiment analysis. Then the processing procedure of the text corpus is detailed, followed by the clustering methodology used to categorize the articles.

#### 2.1.1 Collection of text-based news articles

Worldwide news media articles related to AVs were collected with the support of the Europe Media Monitor (Europe Media Monitor, no date). A set of keywords (in 6 languages – English, French, German, Spanish, Portuguese and Italian) was defined based on a literature review, with the objective of identifying articles related to AVs. To identify articles which were not in one of these 6 languages, a translation of the English keywords was used instead. The full list of keywords used for gathering these articles is available in the annex.

In total, 45,159 electronic news articles were found and collected during the period from March to November 2019 from 152 countries in the world. From this set, exclusively EU-27 + UK news articles were selected, totalling 19,540 text articles. The articles were available in 22 European languages, with the majority of them being in English (6762), German (5518), Italian (2889) and French (1553).

#### 2.1.2 Sentiment analysis

Sentiment analysis or opinion mining is “the computational study of people’s opinions, sentiments, emotions, appraisals, and attitudes towards entities such as products, services, organizations, individuals, issues, events, topics, and their attributes” (Liu, 2015, Zhang et al., 2018).

The sentiment analysis approach used for this work is based on machine learning using a model that, based on annotated texts, is able to learn to classify sentiment into the positive, negative and neutral classes (Balahur, 2016). Examples for sentiments in sentences are:

• Positive sentiment sentence: “Automated vehicles are safe.”
• Negative sentiment sentence: “Automated vehicles are unreliable.”
• Neutral sentiment sentence: “Automated vehicles are vehicles that drive themselves.”

The approach employs a classification model that was trained on labelled tweets and news articles (Barriere and Balahur, 2020), using as features Boolean representations of n-grams, on the support vector machines implementation in Weka (Frank et al., 2016). The performance of the classifier is around 70% F1 score for “native” languages (English, Italian, Spanish, German, French) – i.e. languages for which training data was available and used in the model. Other languages are translated to English for the sentiment analysis, with a slight drop in performance due to the extra step.

#### 2.1.3 Data processing and clustering

Processing of the corpus of the text is necessary to ensure consistency and robustness to the analysis. As a first step, the non-English text articles were translated to English, as a way of carrying out the analysis in one language.

Then, the text data was lemmatized, which performs a normalization of words that carry similar meaning in a sentence, chosen by convention (e.g. nominative singular for nouns, infinitive for verbs). To preserve the meaning of some specific terms, no stemming was carried out. Instead, words and N-grams (terms composed of multiple words) which were relevant to the topic were recoded into one term representing them (e.g. “self driving car” and “autonomous vehicle” were recoded into autonomous_vehicle. The full list of recoded words is available in the annex).

Moreover, a list of stopwords was created, which contains terms that should not be considered in the clustering, as they do not add information about the autonomous car topic. These consist mainly of names (e.g. country, person, company), dates and numbers, as well as commonly used words in English (such as “the”, “a”, “an”, “in”).

For the last step of the processing, the article words were transformed into features (a numerical representation of words) using the term frequency - inverse document frequency (TF-IDF) method (Juan Ramos, 1975). The TF-IDF method assigns importance to words according to their frequency of appearance in individual documents and offsets this importance based on the frequency of the word in the whole group of documents.

In total, 1000 features were chosen to represent the whole news articles dataset. Words were only considered when present in at least 100 different articles (minimum document frequency) to exclude rare words and at maximum 5400 articles (maximum document frequency) to exclude very frequent words.

Finally, the articles were clustered according to their features, using the K-means (Jain, 2010) methodology. The number of clusters chosen was 4, defined by plotting the cluster inertia as a function of the number of clusters and finding the point with the highest curvature, also known as the elbow method (Marutho et al., 2018). These clusters are given a topic name and description, which are based on their prevalent words.

To represent the most prevalent cluster per country, the number of articles for each country was normalized according to the size of the cluster they belong to, ensuring that large clusters did not biased the analysis.

For the news data processing and clustering, Python 3.7 was used, with the scikit-learn (scikit-learn:Machine Learning in Python, no date) and NLTK libraries (Bird, Steven, 2009).

### 2.2 Segmentation of European citizens

Citizens’ opinions were gathered through the special Eurobarometer Survey 496 on “Expectations and concerns of connected and autonomous driving” (European Commission, 2020b), simultaneously to the Eurobarometer survey 495 on “Mobility and Transport” (European Commission, 2020a). A total of 27,565 citizens from the EU-27+UK were interviewed face to face in their mother tongue during two weeks in September 2019. The aim of the Eurobarometer survey 496 was to measure public awareness and attitudes towards AVs. It showed that European citizens are not yet ready to transition to the use of autonomous cars, with the majority of respondents not feeling comfortable in the presence of AVs on the roads, and a minority being willing to purchase such a car. These results demonstrated that there is a need to deepen the opinions expressed by European citizens in order to understand how to address this lack of trust towards autonomous cars. Based on the data of both Eurobarometer surveys, we derived different profiles based on general attitudes towards AVs to explore the specific characteristics that belong to different opinions. Especially, we propose a Latent Class analysis to identify these profiles, followed by inferential statistics to highlight differences among the profiles, based on socio-demographic variables and opinions towards AVs. The analysis did not consider cross-cultural equivalence between the different countries included in the survey, but rather focused on the general opinion expressed (for reference and use, the composition of the different profiles according to the countries is included in the Annex).

#### 2.2.1 Latent class analysis

In order to identify different profiles of citizens according to their acceptance towards AVs, a Latent Class Analysis (LCA) was performed using the R package poLCA developed by (Linzer and Lewis, no date). LCA has been applied in different domains as psychology, education, sociology and in behavioral studies (Linda M. Collins, 2010) but its application in the transport field is still limited (Molin et al., 2016). It has been used to investigate environmental preferences towards cycling (Mertens et al., 2016, Verhoeven et al., 2018), to identify different groups of multimodal users (Molin et al., 2016), to explore historical reasons for owning a car (Araghi et al., 2017), to characterize bicycle crash patterns (Prati et al., 2017), to examine how life courses related to modal behaviour (Delbosc and Nakanishi, 2017) or to search barriers in adopting mobility as a service (Alonso-González et al., 2020).

LCA is a finite mixture modelling that can be used to analyse the structure of relationships among categorical variables (McCutcheon, 1987). This person-centred approach posits that there is an underlying unobserved categorical variable that divides a population in latent classes or clusters. At the difference of traditional cluster analysis methods (i.e. hierarchical or k-means clustering) that creates a measure of dissimilarity between individuals to determine the underlying subgroup structure, LCA methods use a probabilistic modelling approach to identify the likely distributions with the data and the likely placement of individuals within those distributions (Kent, Jensen and Kongsted, 2014). On the other hand, a key advantage of LCA over other traditional clustering methods is the implementation of fit statistics to identify the appropriate number of clusters (Vermunt, 2004).

#### 2.2.2 Cluster number selection

To identify the number of classes to retain, different models were estimated, each specifying different number of classes, starting with one class and continuing to increase in order to determine if the set of available fit statistics point to a class to retain. To select the best model fit, the value of the likelihood ratio chi square statistic (L^2^), the Bayesian information criterion (BIC) (G, 1978), the Akaike information criterion (AIC) (Akaike, 1974), the consistent AIC (CAIC) (Bozdogan, 1987) and the adjusted BIC (aBIC) (Sclove, 1987) were estimated. Lower values are preferable for those fit statistics. Finally, the solution was assessed to ensure that the selected model yields latent classes that are both meaningful and distinguishable from each other (Linda M. Collins, 2010).

For the analysis, different variables from the Eurobarometer survey expressing different aspects of the acceptance towards AVs were selected:

– If you had the opportunity, would you be ready to use the following vehicle types: fully automated? *(Scale from* 1 *to 4 and don’t know)*
– To what extent would you feel comfortable or not travelling in a fully automated vehicle under the following conditions: without the supervision of a human operator? *(Scale from 1 to 4 and don’t know)*
– Please tell me to what extent are you in favour of or opposed to each of the following: The deployment of fully automated vehicles on our roads? *(Scale from 1 to 4 and don’t know)*
– In the last twelve months, have you heard, read or seen anything about automated vehicles? *(Items: Yes – No – Don’t know)*
– Would you ever consider purchasing an automated vehicle? *(Items: Yes – No – don’t know)*

For the purpose of the analysis, the variables ranging from 1 to 4 and including *don’t know* were converted in variables scaling from 1 to 2 and *don’t know*. This choice was motivated by two reasons: the number of classes estimated was too high to reach a best model fit; and the difficulty to interpret the different classes according to the concepts of latent class separation and homogeneity (Linda M. Collins, 2010).

Table 1presents the fit of consecutive model starting with a model with one class up to a model with eight classes. The different fit indexes suggest that the sixth, the seventh and the eighth cluster solutions are optimal. While the likelihood-ratio chi-squared together with the AIC are still decreasing at the eighth cluster, the BIC is at its lowest value on the sixth, and the ABIC together with the CAIC at the seventh. Monte-Carlo simulations suggest that BIC criterion is a better indicator of performance (Nylund et al., 2007), and in accordance to guidelines, the number of clusters with the lowest BIC should be selected. The 6-cluster solution is the most statistically parsimonious solution. However 5-cluster and 7-cluster solutions were also investigated, to observe how the participants are distributed in these other sets of clusters. For the 6-cluster and 7-cluster solutions, some identified groups are small and represent a low proportion of the population surveyed (N < 1000; 3.5%). Therefore, the 5-cluster solution is preferred over the other ones^2^.

**Table 1:** Model fit of the latent class analysis models.

Model nb	Nb of Clusters	LL	L^2^	BIC	AIC	ABIC	CAIC	Npar	df
Model 1	1-Cluster	−108476.67	36109.1346	217055.6	216973.3	217023.8	217065.6	10	232
Model 2	2-Cluster	−93221.48	5598.7541	186657.7	21,186,485	186590.9	186678.7	21	221
Model 3	3-Cluster	−91161.63	1479.0455	182650.4	182387.3	182548.7	182682.4	32	210
Model 4	4-Cluster	−90947.26	1050.3177	182334.2	181980.5	182197.5	182377.2	43	199
Model 5	5-Cluster	−90715.57	586.9361	181983.3	181539.1	181811.6	182037.3	54	188
Model 6	6-Cluster	−90642.26	440.3186	181949.1	181414.5	181742.5	182014.1	65	177
Model 7	7-Cluster	−90587.37	330.5223	181951.8	181326.7	181710.3	182027.8	76	166
Model 8	8-Cluster	−90554.34	264.4735	181998.2	181282.7	181721.7	182085.2	87	155

LL = final log-likelihood of the model.

Npar = number of parameters.

df = degrees of freedom.

Nb = number.

Finally, an analysis to understand the differences among profiles was performed using 79 variables from the Eurobarometer survey 496 and additional data retrieved from the Eurobarometer survey 495 (European Commission, 2020a, European Commission, 2020b). A chi-square test was performed, followed by the Cramer’s V test since chi-square results are very sensitive to the sample size, and do not inform on the strength of the relation of the variables compared. In absence of a strong hypothesis regarding both populations studied, the guideline proposed by (Cohen, 1988) for social science studies is used. For a Cramer’s coefficient above 0.5 we consider a relation as strong, above 0.3 up to 0.5 as moderate and from 0.1 up to 0.3 as weak. Relations below the lower threshold of 0.1 are considered as too weak. For the purpose of the analysis, the variables scaling from 1 to 4 and including *don’t know* were converted in nominal variables (i.e. 1–2 and *don’t know*). Finally, the Vcd package (Hornik et al., 2017) was used to perform the chi-square and Cramer’s V tests.

## 3 Results

The present section outlines the results from the analysis of worldwide media articles (sentiment analysis) and the main findings from the survey concerning people’s attitudes towards connected and autonomous cars.

### 3.1 Media analysis

**Fig. 1:**
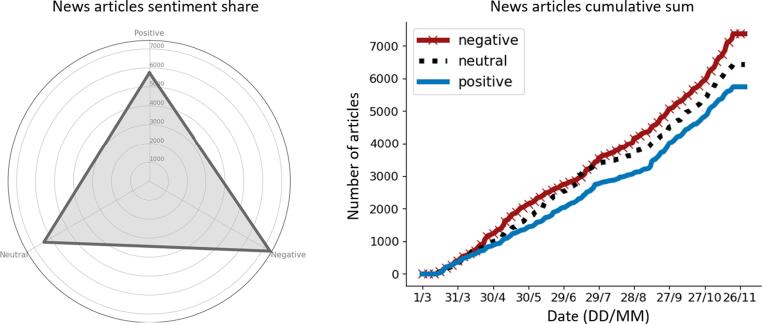
Share of sentiment for news article dataset (left). Overall number of articles per sentiment and cumulative sum for the whole collection period (right). Period: March to November 2019.

As a first step, the overall number of articles reflecting positive, negative and neutral sentiments was analysed, as well as the evolution over time using cumulative sums (Fig. 1). This allows us not only to understand which sentiment prevails, but also to visualize if there are particular events (such as incidents or new technologies developed) or periods in which there is great rise of a given sentiment. The predominant sentiment is negative over the period of analysis, exhibiting smooth increases in the numbers of articles for certain dates. Neutral sentiment articles are second place and also exhibit smooth jumps in numbers. The positive sentiment articles are less present and follow a nearly linear trend with a lower slope.

The news articles can refer to any subject, some of which are more likely to contain negative words and sentences, which can be misleading when trying to capture the way media depicts autonomous cars. Therefore, subdividing the dataset in categories can help to understand and if necessary, exclude any unwanted subjects.

The collection of articles was divided in four clusters, according to the most common words mentioned in the articles. The clusters were named and descriptions were given according to their most prevalent words. The cluster topic names and descriptions are the following:

1) *Development:* cluster related to news on the development of autonomous cars and components, including hardware and software, research, data collection;
2) *Test and Safety:* news articles about testing and safety related information;
3) *Mobile Infrastructure:* contains entries that discuss connectivity and mobile infrastructure related to autonomous cars;
4) *Market:* news articles which contain information about the market, such as startups, new companies, stock news, mergers, and investments and funds.

The top ten words (closest features to the cluster centers) and the number of articles for each cluster are shown in Table 2. To calculate the top ten terms for each cluster, the ten features closest to each cluster center were selected and mapped back to their respective words. Assessing these words is a way of verifying the robustness of the analysis (e.g. if the words are relevant to the topic and if the clusters are well separated and defined).

**Table 2:** Top words and total number of articles per cluster.

Cluster	Top 10 words	Number of articles
1. Development	mobility, transport, human_driver, research, job, develop, cooperation, model, autonomous_driving, data	14113
2. Test and Safety	autonomous_vehicle, test, autonomous_driving, human_driver, safety, develop, transport, automate, sensor, research	2943
3. Mobile Infrastructure	5G, mobile, network, telecom, generation, operator, speed, internet, fast, communication	1037
4. Market	investment, autonomous_vehicle, autonomous_driving, value, opportunity, fund, initial_public_offering, price, cost, range	1447

The results show that cluster number 1, *development*, contains by far the highest number of articles, followed by *test and safety,* meaning that most AV related text news articles tend to focus on these two topics.

**Fig. 2:**
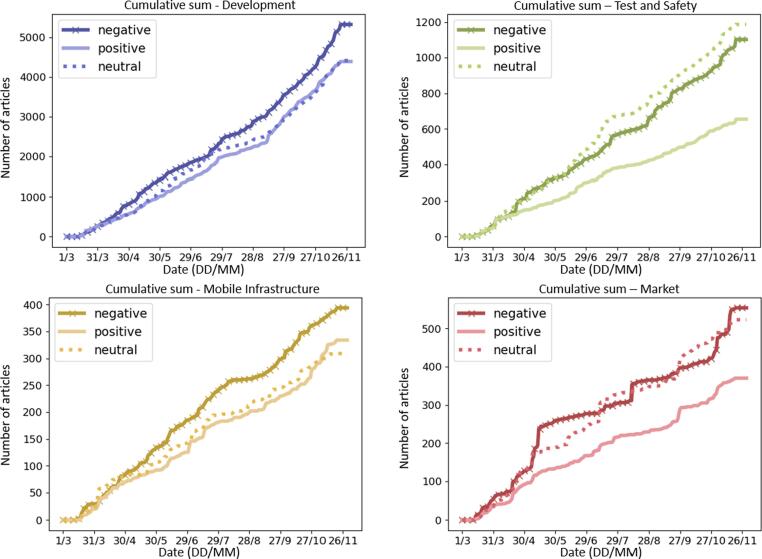
Cumulative sum of articles per sentiment and clusters (from March to November 2019).

We then look at the cumulative sum curves for each cluster (Fig. 2), where it is visible that the clusters have different proportions between sentiments, when compared to the overall numbers from Fig. 1, with the *market* and *test and safety* cluster groups exhibiting a higher proportion of negative sentiments, showing that there is a different tendency in terms of the sentiment.

Moreover, Fig. 2 shows that the groups with the highest imbalance between sentiments are the ones that contain the steepest curves for the whole collection period, which is the case for *market* and *test and safety.* The other two groups, *development* and *mobile infrastructure* show smoother curves, with smaller differences in numbers between the sentiments.

**Fig. 3:**
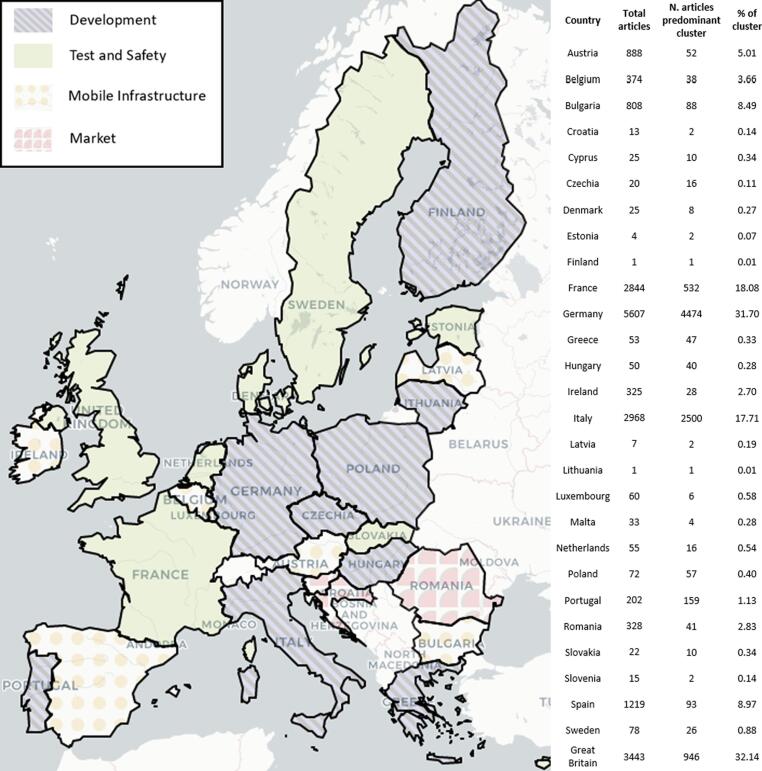
Predominant cluster per country in the analysis, number of articles per country and per predominant cluster, and the ratio of the articles with respect to the cluster.

Finally, the most dominant cluster per country is shown in Fig. 3, along with the total number of articles per country, the total number of articles belonging to the predominant cluster and the percentage of these articles in relation to the cluster (e.g. in Portugal there were 202 articles, out of which 159 belong to cluster 1-*development*, and they make for 1.13% of the whole *development* cluster). It is visible that the *development* and *test and safety* clusters are the most present, given that they contain the highest numbers of articles. Nonetheless, the two smallest clusters, *mobile infrastructure* and *market* still form the majority of news articles for some countries.

### 3.2 Citizens’ opinions

#### 3.2.1 Sample characteristics

The sample was composed of 45% of men and 54% of women, aged 15 years and older (average age is 51). As a transport mode, they primarily used the car (51%), followed by walking (18%) and urban public transport (17%). Additionally, 46% of them would be willing to pay more to improve their mobility.

A majority of the respondents of the survey, six in ten, declared to have heard, read or seen something about AVs in the previous twelve months. However, three quarters said they would not feel comfortable travelling in an AV without any human supervision, nor being in the presence of AVs on the roads, regardless of how they are travelling: as pedestrians (63%), cyclists, motor scooter riders (63%), motorcyclists (62%), travellers in conventional cars (55%) or passengers of autonomous cars (57%).

Less than half of the participants said to be in favour of the deployment of AVs and half of them would never buy an AV, while only less than three in ten say they would purchase one after they have seen them being used by others. Less than half of people stated to be ready to purchase an AV (44%), and especially if they would see them used before (26%).

Diverse opinions are expressed in relation to the possible impacts that AVs could have. Among the expected impacts, respondents think that AVs will reduce the need for professional drivers and take over their jobs (32%), but that they will also reduce accidents (26%) and travel stress (24%).

#### 3.2.2 Results of the latent class analysis

**Fig. 4:**
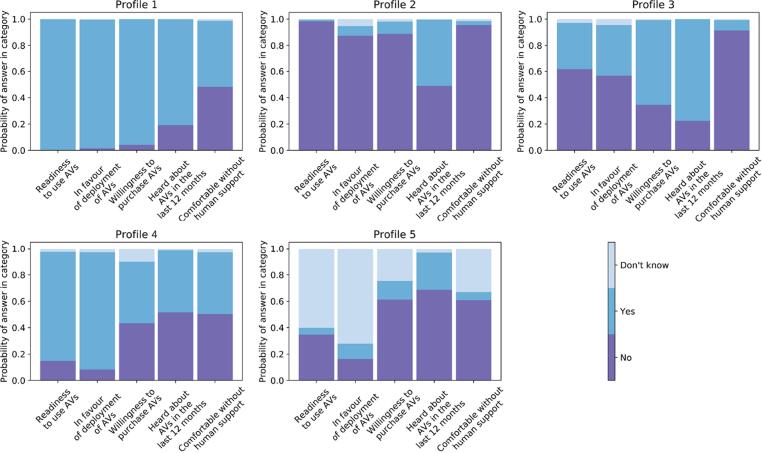
Parameter estimates for latent class analysis model with five profiles.

Respondents were divided in 5 profiles based on their responses towards five variables related to the acceptance towards AVs. Fig. 4 presents, for each profile, the estimated probabilities of answering *Yes*, *No* or *Don’t know*, for each of the variables.

The overall profile characteristics are as follows:

**Profile 1 (N = 7760, 28.2%).** In this profile, there is a very high probability that respondents are in favour of the deployment of AVs, and willing to use and buy them. They are also more likely to have read and heard about AVs during the past year before the interview, and they are the most comfortable with the idea of travelling in an AV without human support.

**Profile 2 (N = 11930, 43.3%).** Citizens belonging to this profile have a negative opinion towards AVs in general, with a higher probability of not being in favour of their deployment, and not willing to use or buy them. Correspondingly, they are also more likely to feel uncomfortable travelling in an AV without human support.

**Profile 3 (N = 3390, 12.3%).** Within this group, individuals are more likely to have heard about AVs in the past year before the interview. They share a higher probability of having negative attitudes towards the use of AVs, and do not feel comfortable travelling in an AV without human support. Nonetheless, they are likely to purchase AVs once these are fully deployed.

**Profile 4 (N = 3228, 11.7%).** Respondents in this profile are more probable to be in favour of the deployment and use of AVs. In addition, they are very likely to feel comfortable travelling in AV without human support.

**Profile 5 (N = 1257, 4.6%).** This profile is composed of less decided citizens, with the highest probability of answering *don’t know* to the questions. Moreover, they share a greater probability to not have read and heard about AVs during the past year before the interview.

#### 3.2.3 Description and analysis of the different profiles

The identified profiles showed significant differences. A chi-square test showed that 100% of the 79 variables used to describe and compare the different profiles were statistically significant. Additionally, the Cramer’s V test demonstrated that 12 variables had a Cohen’s coefficient below the threshold of 0.1, hence being insufficiently strong to be considered in the analysis. Therefore, 67 variables (out of the original 79) were finally used for the analysis.

For a better understanding, a summary of the prevalent profiles is presented according to their socio-demographic characteristics, mobility habits and attitudes, opinions and beliefs towards AVs. Additionally, a table containing the percentage of the responses for each of the variables and profiles is included in the Annex.

**Socio-demographic characteristics and mobility habits**

Table 3 provides a summary of the socio-demographic characteristics and mobility habits of the different profiles. It can be seen that citizens belonging to profile 1 are younger when compared to the other groups, live in a large household (with more than 4 people), and a large majority of them use the internet almost every day. Differently, people within profiles 2 and 5 are generally older than 55 years, are women, and do not use internet.

**Table 3:** Summary of the socio-demographic characteristics and transport habits of the different profiles

**Profile 1 (N = 7.760, 28.2%)**
• Younger • Large household (≤4) • High share of people using internet almost every day • Highest share of people owning a driving license • Highest share of people using car as main transport mode • Mostly willing to pay more to improve their mobility
**Profile 2 (N = 11.930, 43.3%)**
• Older • Mostly women • High share of people never using internet • High share of people not owning a driving license • High share of people using PT and walking as main transport mode • Mostly not willing to pay more to improve their mobility
**Profile 3 (N = 3.390, 12.3%)**
• High share of people owning a driving license • High share of people using car as main transport mode
**Profile 4 (N = 3.228,11.7%)**
• High share of people not owning a driving license • High share of people using PT and walking as main transport mode
**Profile 5 (N = 1.257, 4.6%)**
• Older • Mostly women • High share of people never using internet • Highest share of people not owning a driving license • Mostly not willing to pay more to improve their mobility

Additionally, citizens in profiles 1 and 3 are very similar with respect to their mobility habits, but in different proportions. People belonging to these profiles own a driving license and their main transport mode is the car. In addition, within profile 1, they are willing to pay more to improve their mobility. A non-negligible part of the citizens belonging to profile 2 and 4 do not own a driving license and mostly use urban public transport and walking as their main transport modes. Profile 5 has the highest rate of citizens not owning a driving license. Finally, most citizens in profiles 2 and 5 are not willing to spend more money to improve their daily mobility.

**Attitudes, opinions and beliefs towards AVs**

Table 4 provides a summary of the attitudes, opinions and beliefs of the different profiles. There are two opposite profiles where opinions towards AVs are clearly defined as positive or negative (respectively profiles 1 and 2), while other profiles depict a more complex picture. Especially, participants in profile 5 differentiate from the other profiles with a higher difficulty to answer the survey, demonstrated by their *don’t know* answers on the different variables assessed.

**Table 4:** Summary of the Attitudes, opinions and beliefs towards AVs of the different profiles

**Profile 1 (N = 7.760, 28.2%)**	
• Experience with driving assistance systems • Associate better their ideas of AVs with different pictures (Car, Shuttle, Truck) • Feel more comfortable transporting their children in an AV (with or without human support) • Feel more comfortable transporting goods in an AV (with or without human support) • Feel comfortable sharing the streets with AVs	• Comfortable with any scenario related to taking back the control of the vehicle • Willing to purchase AVS if affordable and after seeing people using them • AVs fit their mobility needs (especially a private vehicle) • More willing to perform activities while in an AV • Stronger beliefs towards AVs • More willing to share private data • Consider all stakeholders to be important for the deployment of AVs
**Profile 2 (N = 11.930, 43.3%)**	
• Not comfortable transporting their children in an AV (with or without human support) • Not comfortable transporting goods in an AV (with or without human support)	• Do not feel comfortable sharing the streets with AVs • Uncomfortable with any scenario related to taking back the control of the vehicle • AVs do not fit their mobility needs • Not willing to share private data
**Profile 3 (N = 3.390, 12.3%)**	
• Experience with driving assistance systems • Do not feel comfortable sharing the streets with AVs • Willing to purchase after seeing people using them	• AVs fit their mobility needs • Not willing to share private data
**Profile 4 (N = 3.228,11.7%)**	
• Feel more comfortable transporting their children in an AV (with or without human support) • Feel more comfortable transporting goods in an AV (with or without human support)	• Feel more comfortable sharing the streets with AVs • Not willing to purchase AVs • Willing to share private data • All stakeholders are important for the deployment of AVs
**Profile 5 (N = 1.257, 4.6%)**	
• No experience with driving assistance systems • Difficulty to associate their ideas of AVs with different pictures (Car, Shuttle, Truck) • Don’t know and don’t think that AVs fit their mobility needs • Don’t know if they feel comfortable transporting their children in an AV (with or without human support)	• Don’t know if they feel comfortable transporting goods in an AV (with or without human support) • Don’t know and don’t feel comfortable sharing the streets with AVs • Don’t know if they are willing to share data • Don’t know which stakeholders are important for the deployment of AVs

Participants belonging to profiles 1 and 3 have more experience with automated and semi-automated driving assistance systems, while the ones within profile 5 expressed the lowest level of experience. People in profiles 1 and 4 would generally feel more comfortable with transporting their children and their goods in an AV. In addition, when it comes to the value of the goods transported, participants within profile 1 are more comfortable to use AVs for the pick-up and delivery of both low-value and high-value goods compared to the other profiles. In opposition, participants belonging to profile 2 would feel less comfortable transporting children or goods with AVs and participants belonging to profile 5 stand by the *don’t know* answers. Regarding the idea of sharing the streets with AVs (i.e. as pedestrians, as cyclists, as motor scooter riders, as motorcyclists, as conventional car drivers, as passenger of an AV), most participants belonging to profile 1 would feel more comfortable regardless of the mode of transport used, while people in profiles 2 and 3 would not feel comfortable. Regarding having the ability of taking back the control of the AVs under different circumstances (i.e. at any point in time, when the vehicle tells that it does not understand the situation, in case of emergency, and to avoid accidents) citizens in profile 1 would feel comfortable under all the circumstances, while participants in profile 2 would not feel comfortable under any of the above-mentioned circumstances.

Regarding the willingness to purchase an AV, the vast majority of participants in profile 1 would buy them if affordable or if they see others using them first. Especially, they think that a private vehicle would fit better their mobility needs. In a lower proportion, people in profile 3 think that AVs would fit their mobility needs and would prefer to use it or would hire an AV for their individual needs. They would also be willing to buy AVs, but only if they see them being used before. Interestingly, while a large share of participants belonging in profile 4 think AVs would fit their mobility needs, a strong majority declared they would not buy them. Finally, in profile 2, most citizens do not think that AVs would fit their mobility needs.

With respect to their willingness to perform other activities while an AV would be driving, participants in profile 1 would be in general more willing to perform some of them (i.e. sleeping, working, entertain themselves, listen to music or radio, look the scenery), while participants in profile 3 would rather pay attention to the road and other vehicles.

Participants within profile 1 have stronger beliefs regarding the impact of AVs at individual and societal levels, when compared to the other profiles. They believe that AVs can reduce accidents, reduce congestion, increase activities while driving, reduce the need for professional drivers, increase accessibility, reduce stress, decrease travel time, reduce emissions, improve driving comfort. Moreover, citizens of profiles 1 and 4 are more willing to share their mobility data with different entities (i.e. road users, public authorities, private companies). In opposition, participants belonging to profiles 2 and 3 are not willing to share their mobility data with other road users in general, and citizens within profile 5 stand by their propensity to answer that they *don’t know.* Finally, citizens from profiles 1 and 4 believe all stakeholders (i.e. private companies, public authorities, the European Union and international organizations) to be important for taking care of the deployment of AVs. Citizens within profile 5 *do not know* which stakeholders should have an important role in the deployment of AVs.

## 4 Discussion and conclusions

### 4.1 Media analysis

For the media article sentiments analysis related to AVs, our results show that the most present sentiment for all clusters is negative. This could be linked to fears of overall impacts that new technologies could have, which could stimulate stronger sentiments, notably more towards the negative side (Anania et al., 2018). From the variety of topics on autonomous cars, some tend to carry more negative messages than others.

One of the groups that contains a majority of negative sentiments, *market,* includes a variety of news articles linked to business activities, stock markets, financial information and employment. Some of these (e.g. news on automation reducing employment) can capture the readers’ attention and negatively influence their acceptance of autonomous cars. Very relevant to the acceptance of autonomous cars, *test and safety* news articles can definitely have an influence on the public, especially if they portray predominantly negative sentiments related to autonomous car accidents. The relative balance between sentiments in the other two groups, *development* and *mobile infrastructure*, leads us to believe that the technology-oriented news articles tend to show fewer negative impacts.

### 4.2 Citizens’ opinions

The results of the segmentation show that the profiles demonstrating more negative attitudes towards AVs (profiles 2 and 3) represent the majority of the whole surveyed population (55.6%). There are two profiles which stand out: profile 1 is composed of enthusiast citizens in favour of the deployment of AVs, while profile 2, the largest one representing 43% of the population interviewed, contains people opposing their deployment. The other profiles, which are less radical, show more complex behaviours, e.g. citizens from profile 5, who mostly don’t know or don’t have a strong opinion, and had not seen or read anything about AVs in the year before the interview. Differently from the literature review, the knowledge about AVs (Bansal et al., 2016, Sener et al., 2019) is not only associated with a positive acceptance towards AVs as in profile 1 but also with a negative one as in profile 3. Interestingly, the difficulty to answer the survey, as in profile 5, is also associated with a lower knowledge towards AVs. Regarding the willingness to purchase AVs, it can be noticed that the two groups which showed a higher level of acceptance towards AVs would not have the same behaviour when it comes to purchasing an AV.

Participants of profile 1 are willing to purchase and use AVs as a main mode of transport, and while members of profile 3 have a negative attitude towards AVs, they would be willing to purchase them if they see people using them first. This result is in line with the findings of (Zhang et al., 2020) and highlights the positive role of social influence on members of this group.

With respect to socio-demographic characteristics, and in line with the literature, positive attitudes towards AVs are associated with a younger age. In addition, the comparison of the different profiles showed that the ones with negative attitudes together with the ones characterised by the *don’t know* answers have higher rates of people who do not own a driving license.

### 4.3 Policy perspective and transferring results

From a policy perspective, the review showed that the narrative is mostly positive, highlighting the benefits and potential that AVs can bring to society, with strategic investments in technological solutions. However, this goes in contrast with the media sentiment, as well as with a large share of the surveyed population, which portray a more reluctant point of view.

When comparing the policy narrative with the media, it is clear that while both tend to have more positive views of technology (with the Development cluster from the media articles showing one of the highest proportions between positive and negative sentiments), there is some divergence with regards to safety (with a positive view coming from policy, and a much more negative perspective coming from media).

With respect to citizens’ opinions (and in contrast with the policy narratives), technological advancements can be of less importance if the citizens are not fully informed about the topic. While the acceptance of AVs is different, depending on the citizen profiles, it is clear that a great share of the population still feels reluctant about the new technology.

While the deeper reasons behind this reluctance in population and media remain to be fully understood, the list of claimed benefits assigned to autonomous cars demonstrates promising disruptive potential. However, the results from the media and survey analyses show that a fruitful transition to autonomous mobility requires much more than solving exclusively technological problems. There should be a societal consensus of its desirability and the willingness to use it, with adequate support to those otherwise undecided or against it. Therefore, to be able to achieve a harmonised and successful transition to autonomous mobility, we encourage policy to adopt a participatory approach and involve citizens both in the development and deployment of future mobility solutions.

As a first step to this approach, we encourage public discussions and opportunities for different users to experience, co-develop and evaluate these new mobility concepts. This could help the public to understand what potential benefits and drawbacks these new AV technologies may bring in practice, while experiencing them firsthand in real-life situations. In this way, citizens could be better prepared to accept these new mobility solutions. These co-creation approaches would also ensure that the new mobility concepts are tailored to their needs, desires and expectations.

In this context, we suggest Living Labs as a suitable way to engage citizens and let them experience AVs in a co-creation environment, sharing knowledge and good practices, and building on each other’s experiences for a better coordination of development activities. Living Labs are “user-centred, open innovation ecosystems based on systematic user co-creation approach, integrating research and innovation processes in real life communities and settings” (What are Living Labs, no date). In this ecosystem, citizens are engaged since the early stages of the innovation development and take an active role in the collaborative development of the new solutions together with other stakeholders from the public sector, academia and industry.

With the evidence collected by directly engaging citizens through a co-creation process, policy initiatives can also be better shaped, taking into account not only the technological but also societal dimensions, which need to support each other to be effective. This would represent a revolution in transport policy development, which is today biased towards the promises of technological innovations whose effects are not yet confronted with the complexity of the urban and regional ecosystems.

Finally, potential changes to mobility must be proposed, going beyond solely new options for covering daily mobility needs. Instead, policymakers should aim for potential changes in mobility patterns, the transport system, and even transformations of cities and rural areas as we know them today.

### 4.4 Conclusions and limitations

Altogether, our findings show that no unanimous view or opinion exists around the concept of autonomous driving. Strong attention is needed when it comes to the claims on autonomous driving benefits - increase in safety, transport accessibility and mobility improvements are among the most frequently mentioned advantages, nonetheless there is no clear evidence that these aspects are recognised and acknowledged by the general public and media. On the contrary, our results show that in many cases, a negative sentiment may be associated to some of these claims and that there is no clear citizens’ view on the enhancement of mobility due to the introduction of this technology. Therefore, the evaluation of new mobility concepts cannot be carried out in a static context, such as a demonstration event lasting for just a few days. The interaction between new technologies and citizens needs to be in place for sufficient time to generate a real impact on digital services and skills, commuting and travelling habits, travel risk perception and the willingness to pay or share transport means.

For the case of AVs, we propose a participatory approach to policymaking, where citizens are engaged and part of the policy development process, being able to understand the implications of the new types of vehicle and the consequences in their daily mobility. In this way, it is possible to develop a new framework which truly benefits the citizen, with less risk of lack of acceptance. To achieve this, we propose a co-creation approach based on Living Labs, where citizens are engaged and can experience AVs in real conditions, before wide-scale deployment, while still being able to have an active role in the development.

Finally, to understand what benefits can realistically be expected from the deployment of AVs, concepts and pilots in specific environments (cities, rural area etc.) should be developed and discussed, tested and evaluated together with stakeholders: citizens, users, car makers, policymakers.

There are some limitations for the different analyses in this article. Since sentiment definition can be subjective and vary from person to person, similar boundaries also exist with automated methods, with divergence in labels. Even though mislabelling can lead to wrong information, it is expected that with large amounts of data, missed labels will have a small impact. Moreover, it is expected that the keywords chosen to find AV related articles might not capture all possible media articles in the subject.

With respect to the citizen analysis, limitations are especially related to the survey design. The amount of topics covered by the survey is limited, in order to keep it with a relatively short length. In addition, the survey can only provide a general snapshot at the moment it was conducted, therefore one should be careful when generalizing results. It may be necessary to consider other methods of citizen consultation and engagement in order to gain a more in depth understanding of public awareness and opinions.

Finally, an overall limitation remains on the link between media and citizen opinions. With the current datasets, it is not possible to analyse the influence that news media has on people’s preferences and vice-versa. Such an analysis can only be carried out with a dedicated study.

## 5 Data availability statement

The news articles data that support the findings of the sentiment analysis of news media part of the study are available from the corresponding author upon reasonable request. The Eurobarometer survey data that support the findings of this part of the study are available in the EU Open Data Portal “https://data.europa.eu/euodp/en/data/dataset/S2231_92_1_496_ENG”

## 6 Code availability statement

The custom code used for this study is available from the corresponding author upon reasonable request.

### CRediT authorship contribution statement

**Fabio Luis Marques dos Santos:** Conceptualization, Methodology, Validation, Visualization, Writing – review & editing. **Amandine Duboz:** Conceptualization, Methodology, Validation, Visualization, Writing – review & editing. **Monica Grosso:** Conceptualization, Methodology, Writing – review & editing. **María Alonso Raposo:** Conceptualization, Methodology, Writing – review & editing. **Jette Krause:** Conceptualization, Methodology, Writing – review & editing. **Andromachi Mourtzouchou:** Conceptualization, Methodology, Writing – review & editing. **Alexandra Balahur:** Data curation, Methodology. **Biagio Ciuffo:** Conceptualization, Methodology, Writing – review & editing, Supervision.

## Declaration of Competing Interest

The authors declare that they have no known competing financial interests or personal relationships that could have appeared to influence the work reported in this paper.

## Footnotes

1 The development of worldwide harmonized regulations concerning the approval and certification of motor-vehicles takes place under the UNECE framework. A specific working part on Automated/Autonomous and Connected Vehicles (GRVA) is in place since late 2018 https://unece.org/transportvehicle-regulations/working-party-automatedautonomous-and-connected-vehicles-introduction.

2 Parameter estimates for latent class analysis model with six and seven clusters solution are presented in Annex.
