# Supplementary material for: An acceptance divergence? Media, citizens and policy perspectives on autonomous cars in the European Union
Source: Transp Res Part A Policy Pract. 2022 Apr;158:224–38. doi: 10.1016/j.tra.2022.02.013 (PMC8988241; doi:10.1016/j.tra.2022.02.013)
Supplement: Supplementary data 1 [file mmc1.docx]

**Annex 1**

1. **Processed bigrams and words with similar meaning:**

**artificial_intelligence** = artificial intelligence , ai

**5G** = 5g network,5g

**autonomous_vehicle** = self driving car, self drive car, autonomous vehicle, self drive vehicle, driverless car, self driving vehicle, autonomous car

**autonomous_driving** = self driving, self drive

**electric_mobility** = electric mobility

**electric_vehicle** = electric vehicle, electric car

**initial_public_offering** = initial public offering

**ride_hail** = ride hail

**human_driver** = driver

**investment** = investor, invest, investment

**smart_mobility** = smart mobility

1. **News articles excerpts for each sentiment and cluster**

Disclaimer: these articles do not represent the opinions of the authors and have been solely included to represent examples of the media used for the analysis

**cluster 1 - Development:**

**neutral**: U.S. seeks input on GM petition to deploy cars without steering wheels - The U.S. National Highway Traffic Safety Administration said Friday it was seeking public comment on General Motors' 15-month-old petition seeking approval to deploy a limited number vehicles on U.S. roads without steering wheels or other human controls.

**negative**: AI will take over your job — maybe - We know artificial intelligence technology will change the future of jobs and the labor market. What we don't know yet is how. In less than a decade, machines could take over 52 percent of the current workload, compared to 29 percent today, according to a report from the World Economic Forum. It's not all bad news, the report claims.

**positive**: Stanford University Launches the Institute for Human-Centered Artificial Intelligence - Stanford University is launching a new institute committed to studying, guiding and developing human-centered artificial intelligence technologies and applications.

**cluster 2 – Test and Safety:**

**neutral**: The Test Fleet of the Future Is Virtual: DRIVE Constellation Now Available - NVIDIA DRIVE Constellation is bringing autonomous vehicle test fleets to the cloud. At the GPU Technology Conference , NVIDIA founder and CEO Jensen Huang announced that the NVIDIA DRIVE Constellation simulation platform is now available.

**negative**: The racism of technology - and why driverless cars could be the most dangerous example yet - There is a rule for dealing with computers: garbage in, garbage out. Put the wrong number of zeroes in your Excel spreadsheet and it will unthinkingly pay your staff pennies on the pound; train a self-driving car to recognise human figures by showing it millions of pictures of white people, and it....

**positive**: THE FUTURE IS BRIGHT A behind the scenes look at how AR can transform the world of journalism - We’ve heard it all before—some new, groundbreaking technology is going to change the way we live and work. In fact, we’ve heard these claims so many times that it’s only natural to feel skeptical of them. While we may still be waiting for self-driving cars to make our gas guzzlers obsolete, amazing....

**cluster 3 – Mobile Infrastructure:**

**neutral**: From London to Tallinn, how five European cities plan to transform mobility with 5G - Super-fast internet speeds grab the headlines when it comes to the potential of 5G – but it also promises to revolutionise urban transport as we know it. – most experts predict it will arrive sometime next year – cities around the world are readying themselves for it....

**negative**: Why is 5G mobile broadband technology such a mystery? The debate around 5G — an embryonic technology that's seemingly poised to replace 4G and meet our ever greater "demands" for mobile data for streaming films, navigating self-driving cars, or getting rich on blockchain — is as much a war about patents as it is about business, security, and, well…....

**positive**: Wipro : Collaborates with IISc for Advanced Research and Innovation in Autonomous Systems, Robotics and 5G - Wipro Limited, a leading global information technology, consulting and business process services company, today announced a strategic partnership with the Indian Institute of Science (IISc), India’s premier public establishment for research and higher education....

**cluster 4 - Market:**

**neutral**: Uber planning to launch IPO in April - Uber Technologies Inc is planning to file for an initial public offering in April, according to people familiar with the matter cited by Reuters. The company will release its public disclosure next month and launch an investor roadshow, the sources said.

**negative**: WARNING LIGHTS Uber raised $1 billion for self-driving cars because it desperately needs the money Quartz - Uber has raised a fresh $1 billion for its driverless cars business from three Japanese investors: Softbank’s Vision Fund, Toyota, and auto-parts maker Denso. The financing establishes Uber’s self-driving cars unit as its own corporate entity, valued at $7.25 billion, the company said in a for a widely anticipated initial public offering.

**positive**: Investors give record support to ride-hailing, EVs, AVs, and Chinese startups - Investors have seen the future when it comes to the almighty car, and it revolves around electrification, driverless vehicles, ride-hailing, and China. Maybe those choices aren’t particularly surprising, but the rate at which backers are throwing money at startups may be.

1. **Keywords used for news articles gathering**

| **English** | **French** | **Spanish** |
| --- | --- | --- |
| self-driving+car | vehicul%+autonom% | vehículo%+autónomo% |
| self-driving+cars | voiture + autonome | vehículos+circularán+sin+conductor |
| automated+vehicle% | voitures + autonomes | veículo%+autônomo% |
| autonomous+vehicle% | voiture + sans + conducteur | veículo%+autônomo+inteligente |
| autonomous-car | voitures + sans + conducteurs | veículo%+robótico% |
| autonomous-cars | voiture sans chauffeur |  |
| driverless+car | voitures + sans + chauffeurs |  |
| driverless+cars | Taxi + autonome |  |
| robot+car | Taxis + autonomes |  |
| robot+cars | Vehicules + autonomes + partages |  |
| automated + car | Vehicule + autonome + partage |  |
| automated + cars | Voiture + autonome + partagee |  |
| automated + vehicles | Voitures + autonomes + partagees |  |
| autonomous + vehicles | Robot + taxi |  |
| driverless + vehicles | Robots + taxis |  |
| driverless + vehicle | navette + autonome |  |
| autonomous vehicle | navettes + autonomes |  |
| automated + shuttles | Taxi + sans + chauffeur |  |
| automated + shuttle | Taxis + sans + chauffeurs |  |
| shared + autonomous + vehicles |  |  |
| shared + autonomous + vehicle |  |  |
| shared + autonomous + car |  |  |
| shared + autonomous + cars |  |  |
| taxi + robot |  |  |
| Taxi + robots |  |  |
| Driverless + taxi |  |  |
| Driverless + taxis |  |  |
| personal rapid transit |  |  |
| personal automated transports |  |  |
|  |  |  |
|  |  |  |
|  |  |  |

| **Italian** | **German** | **Portuguese** |
| --- | --- | --- |
| auto+a+guida+autonoma | automatisches+Fahren | veículo%+autônomo% |
| auto+senza+conducente | automatiserede+køretøjer | carro%+autônomo% |
| auto+senza+guidatore | automatisierte%+Fahren | veículo%+autónomo% |
| autoveicol_+senza+conducente | autonom+fahrendes+Fahrzeug | veículo%+robótico% |
| autovettur_+autonom_ | autonome%+e-fahrzeug | veículo%+sem+motorista% |
| veicol_+senza+conducent_ | autonomem+Fahren | carro%+robótico% |
|  | selbstfahrende%+autos | veículo%+autoconduzido% |
|  | selbstfahrende%+Fahrzeug | veículo%+sem+condutor% |
|  | Roboter+Auto | carro%+sem+condutor% |
|  | Roboter+Fahrzeug | conduç%+autónoma% |
|  | automatisches+Fahrzeug | conduç%+autônoma% |
|  | automatisches+KFZ | robô%+táxi% |
|  | automatisches+Auto | transporte%+autônomo% |
|  | automatisches+Automobil | transporte%+autónomo% |
|  | Fahrerlose+Fahrzeuge | táxi%+sem+condutor% |
|  | Fahrerloses+Fahrzeug |  |
|  | automatische+Shuttles |  |
|  | automatisches+Shuttle |  |
|  | autonomes+carsharing+Fahrzeug |  |
|  | autonomes+carsharing |  |
|  | autonome+carsharing+Fahrzeuge |  |
|  | Fahrerloses+Ruftaxi |  |
|  | Roboter+Taxi |  |
|  | Fahrerloses+Taxi |  |
|  | Fahrerlose+Taxis |  |
|  | Fahrroboter |  |
|  | Car+sharing |  |
|  | Autopilot |  |
|  |  |  |
|  |  |  |

**Annex 2**

1. **Parameter estimates for latent class analysis model with six clusters**

|  |  | **Cluster 1** | **Cluster 2** | **Cluster 3** | **Cluster 4** | **Cluster 5** | | **Cluster 6** | |
| --- | --- | --- | --- | --- | --- | --- | --- | --- | --- |
| **Latent Class membership probabilities** | | 0.0219 | 0.1041 | 0.1475 | 0.2847 | 0.0339 | 0.3825 | |  |
|  |  | **Item response probabilities** | | | | | | |  |
| **Readiness to use Avs** | **No** | 0.1539 | 0.1656 | 0.6983 | 0.0093 | 0.3784 | 0.9865 | |  |
|  | **Yes** | 0.375 | 0.8084 | 0.3017 | 0.9894 | 0.0193 | 0.0049 | |  |
|  | **DK** | 0.4711 | 0.0259 | 0 | 0.0013 | 0.6023 | 0.0086 | |  |
| **Agreement with the deployment of Avs** | **Not in favour** | 0.27 | 0.0762 | 0.6065 | 0.022 | 0.1223 | 0.8775 | |  |
|  | **In Favour** | 0.3301 | 0.8978 | 0.3659 | 0.9746 | 0.0793 | 0.0625 | |  |
|  | **DK** | 0.3999 | 0.026 | 0.0276 | 0.0033 | 0.7984 | 0.06 | |  |
| **Willingness to purchase Avs** | **Yes** | 0.5315 | 0.3921 | 0.5773 | 0.9296 | 0.0496 | 0.0812 | |  |
|  | **No** | 0.2815 | 0.4923 | 0.4227 | 0.0652 | 0.7026 | 0.8975 | |  |
|  | **DK** | 0.187 | 0.1155 | 0 | 0.0052 | 0.2479 | 0.0213 | |  |
| **Heard about Avs in the last 12 months** | **No** | 0.3646 | 0.5635 | 0.2576 | 0.2114 | 0.7843 | 0.4961 | |  |
|  | **Yes** | 0.6184 | 0.4209 | 0.7424 | 0.7863 | 0.1811 | 0.498 | |  |
|  | **DK** | 0.017 | 0.0156 | 0 | 0.0023 | 0.0346 | 0.0059 | |  |
| **Comfortability without human support** | **Not comfortable** | 0.836 | 0.4812 | 0.9083 | 0.4899 | 0.5185 | 0.9568 | |  |
|  | **Comfortable** | 0.0712 | 0.4872 | 0.0858 | 0.4962 | 0.0624 | 0.0277 | |  |
|  | **DK** | 0.0928 | 0.0316 | 0.0059 | 0.0139 | 0.4191 | 0.0155 | |  |

1. **Parameter estimates for latent class analysis model with seven clusters**

|  |  | **Cluster 1** | **Cluster 2** | **Cluster 3** | **Cluster 4** | **Cluster 5** | **Cluster 6** | **Cluster 7** |
| --- | --- | --- | --- | --- | --- | --- | --- | --- |
| **Latent Class membership probabilities** | | 0.0322 | 0.0769 | 0.3719 | 0.0928 | 0.0368 | 0.2835 | 0.1059 |
|  |  | **Item response probabilities** | | | | | | |
| **Readiness to use Avs** | **No** | 0.1355 | 0.112 | 0.9745 | 0.6818 | 0.3914 | 0.0234 | 0.7127 |
|  | **Yes** | 0.3536 | 0.8578 | 0.0182 | 0.3182 | 0.0195 | 0.9744 | 0.2873 |
|  | **DK** | 0.5109 | 0.0302 | 0.0072 | 0 | 0.5891 | 0.0022 | 0 |
| **Agreement with the deployment of Avs** | **Not in favour** | 0.2505 | 0.0791 | 0.9345 | 0.6612 | 0.1328 | 0.0189 | 0.2969 |
|  | **In Favour** | 0.3353 | 0.8885 | 0 | 0.2995 | 0.0859 | 0.9775 | 0.7031 |
|  | **DK** | 0.4142 | 0.0324 | 0.0655 | 0.0392 | 0.7813 | 0.0036 | 0 |
| **Willingness to purchase Avs** | **Yes** | 0.4686 | 0.4571 | 0.0723 | 1 | 0.0516 | 0.8933 | 0.1 |
|  | **No** | 0.3618 | 0.4159 | 0.9081 | 0 | 0.6907 | 0.092 | 0.8769 |
|  | **DK** | 0.1695 | 0.127 | 0.0196 | 0 | 0.2577 | 0.0148 | 0.0231 |
| **Heard about Avs in the last 12 months** | **No** | 0.345 | 0.7334 | 0.4875 | 0.264 | 0.8001 | 0.2032 | 0.3465 |
|  | **Yes** | 0.6436 | 0.2372 | 0.5068 | 0.7352 | 0.1621 | 0.7958 | 0.6528 |
|  | **DK** | 0.0115 | 0.0294 | 0.0057 | 0.0008 | 0.0377 | 0.001 | 0.0006 |
| **Comfortability without human support** | **Not comfortable** | 0.8358 | 0.394 | 0.9582 | 0.9121 | 0.512 | 0.4947 | 0.8658 |
|  | **Comfortable** | 0.0763 | 0.5692 | 0.0276 | 0.0812 | 0.0638 | 0.491 | 0.1196 |
|  | **DK** | 0.0879 | 0.0367 | 0.0142 | 0.0067 | 0.4242 | 0.0143 | 0.0146 |

1. Country composition for the 5 profiles

| **Count of Profiles** | **Column Labels** |  |  |  |  |  |
| --- | --- | --- | --- | --- | --- | --- |
| **Row Labels** | **1** | **2** | **3** | **4** | **5** | **Grand Total** |
| Austria | 30.10% | 43.33% | 8.82% | 13.24% | 4.51% | 100.00% |
| Belgium | 36.07% | 37.21% | 14.50% | 11.55% | 0.67% | 100.00% |
| Bulgaria | 22.47% | 38.42% | 3.99% | 19.75% | 15.37% | 100.00% |
| Croatia | 34.69% | 39.52% | 11.79% | 10.24% | 3.77% | 100.00% |
| Czech Republic | 24.40% | 46.73% | 11.90% | 12.30% | 4.66% | 100.00% |
| Denmark | 36.98% | 37.38% | 12.43% | 11.13% | 2.09% | 100.00% |
| Deutschland Est | 28.12% | 50.84% | 11.73% | 8.01% | 1.30% | 100.00% |
| Deutschland West | 29.86% | 44.36% | 15.56% | 7.78% | 2.43% | 100.00% |
| Estonia | 21.79% | 45.44% | 13.15% | 13.54% | 6.08% | 100.00% |
| Finland | 23.36% | 49.56% | 13.84% | 10.79% | 2.45% | 100.00% |
| France | 28.13% | 48.01% | 14.61% | 7.16% | 2.09% | 100.00% |
| Greece | 26.95% | 54.79% | 10.27% | 6.22% | 1.78% | 100.00% |
| Hungary | 24.06% | 43.66% | 11.68% | 18.22% | 2.38% | 100.00% |
| Ireland | 32.15% | 38.48% | 10.39% | 13.45% | 5.54% | 100.00% |
| Italia | 25.83% | 49.32% | 12.09% | 8.09% | 4.68% | 100.00% |
| Latvia | 20.68% | 46.72% | 15.90% | 11.93% | 4.77% | 100.00% |
| Lithuania | 19.98% | 49.95% | 16.18% | 10.39% | 3.50% | 100.00% |
| Luxembourg | 30.36% | 43.06% | 17.26% | 5.75% | 3.57% | 100.00% |
| Malta | 25.74% | 37.82% | 7.72% | 12.28% | 16.44% | 100.00% |
| Netherlands | 37.37% | 33.08% | 22.12% | 6.96% | 0.48% | 100.00% |
| Poland | 24.00% | 37.50% | 8.00% | 21.10% | 9.40% | 100.00% |
| Portugal | 25.34% | 48.64% | 5.44% | 13.59% | 6.99% | 100.00% |
| Republic of Cyprus | 36.11% | 41.47% | 8.73% | 8.13% | 5.56% | 100.00% |
| Romania | 27.05% | 31.54% | 11.04% | 23.34% | 7.03% | 100.00% |
| Slovakia | 26.32% | 47.07% | 9.61% | 9.51% | 7.49% | 100.00% |
| Slovenia | 33.00% | 41.00% | 16.70% | 7.90% | 1.40% | 100.00% |
| Spain | 23.90% | 48.71% | 12.95% | 9.66% | 4.78% | 100.00% |
| Sweden | 36.05% | 35.56% | 15.50% | 11.92% | 0.97% | 100.00% |
| United Kingdom | 28.64% | 46.34% | 10.85% | 9.51% | 4.66% | 100.00% |
| **Grand Total** | **28.15%** | **43.28%** | **12.30%** | **11.71%** | **4.56%** | **100.00%** |

1.
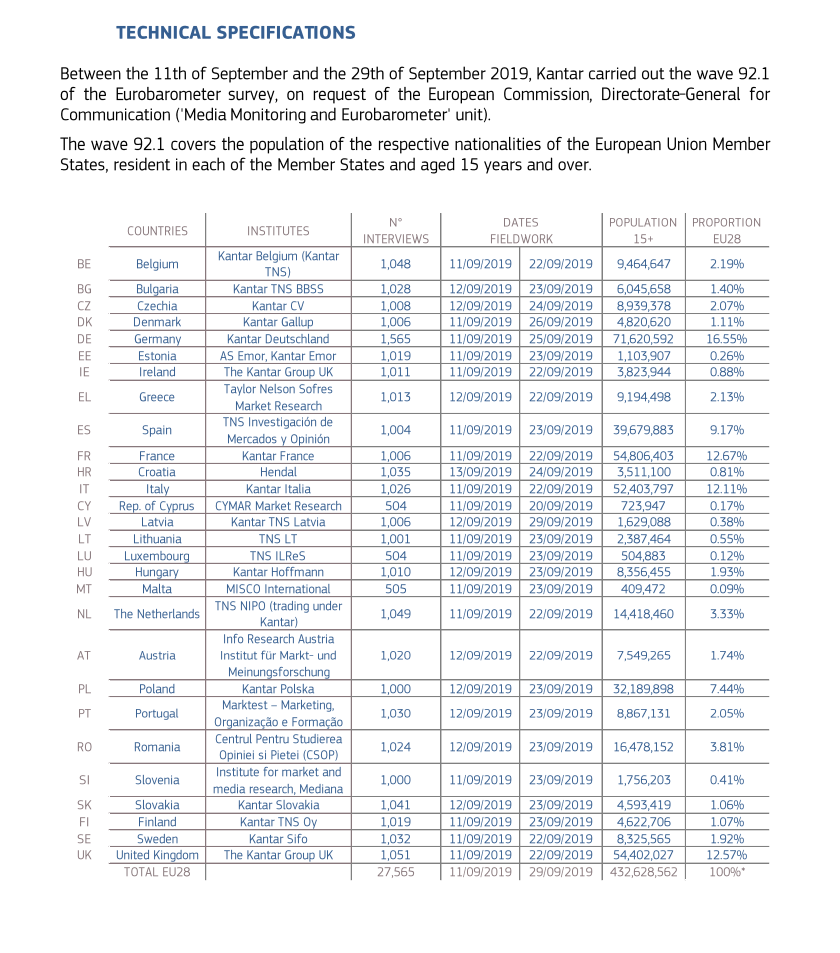
Survey interviews sample breakdown and periods, as reported in the “Special Eurobarometer 496: Expectations and Concerns from a Connected and Automated Mobility”
2. Results of the analysis

| **Attitudes towards automated driving** | | | | | | |
| --- | --- | --- | --- | --- | --- | --- |
|  |  | **Profile 1** | **Profile 2** | **Profile 3** | **Profile 4** | **Profile 5** |
| **Comfortable with transporting children in an AV** | | | | | | |
| **With the supervision of a human operator** | Not Comfortable | 13% | 60% | 36% | 21% | 30% |
|  | Comfortable | 84% | 36% | 62% | 75% | 38% |
|  | Don't know | 2% | 3% | 2% | 5% | 32% |
| **With the supervision of a remote human support** | Not Comfortable | 39% | 89% | 78% | 42% | 52% |
|  | Comfortable | 59% | 8% | 19% | 53% | 14% |
|  | Don't know | 3% | 3% | 2% | 5% | 34% |
| **Without the supervision of a human operator** | Not Comfortable | 57% | 94% | 92% | 55% | 59% |
|  | Comfortable | 40% | 3% | 5% | 39% | 5% |
|  | Don't know | 3% | 3% | 2% | 6% | 36% |
| **Comfortable with transporting your goods in an AV** | | | | | | |
| **With the supervision of a human operator** | Not Comfortable | 5% | 42% | 17% | 11% | 17% |
|  | Comfortable | 94% | 54% | 82% | 86% | 52% |
|  | Don't know | 1% | 4% | 1% | 2% | 31% |
| **With the supervision of a remote human support** | Not Comfortable | 13% | 61% | 39% | 20% | 31% |
|  | Comfortable | 86% | 35% | 60% | 78% | 36% |
|  | Don't know | 1% | 4% | 1% | 3% | 33% |
| **Without the supervision of a human operator** | Not Comfortable | 25% | 71% | 56% | 30% | 39% |
|  | Comfortable | 74% | 25% | 42% | 67% | 27% |
|  | Don't know | 1% | 4% | 1% | 3% | 35% |
| **Comfortable with the pick-up and delivery of goods** | | | | | | |
| **Low-value goods** | Not Comfortable | 7% | 55% | 29% | 15% | 24% |
|  | Comfortable | 92% | 39% | 68% | 81% | 39% |
|  | Don't know | 1% | 5% | 3% | 3% | 37% |
| **High-value goods** | Not Comfortable | 21% | 71% | 52% | 27% | 33% |
|  | Comfortable | 77% | 24% | 45% | 69% | 29% |
|  | Don't know | 1% | 5% | 3% | 4% | 38% |
| **Sharing the streets with AVS using different modes of transport** | | | | | | |
| **Pedestrian** | Not Comfortable | 30% | 85% | 73% | 37% | 52% |
|  | Comfortable | 68% | 13% | 25% | 61% | 21% |
|  | Don't know | 1% | 2% | 2% | 3% | 28% |
| **Cyclist** | Not Comfortable | 37% | 86% | 78% | 42% | 52% |
|  | Comfortable | 61% | 9% | 20% | 53% | 15% |
|  | Don't know | 2% | 5% | 3% | 5% | 33% |
| **Scooter rider** | Not Comfortable | 38% | 83% | 76% | 42% | 48% |
|  | Comfortable | 57% | 7% | 17% | 48% | 13% |
|  | Don't know | 6% | 11% | 7% | 10% | 39% |
| **Motorcyclist** | Not Comfortable | 36% | 82% | 76% | 40% | 48% |
|  | Comfortable | 58% | 7% | 17% | 49% | 13% |
|  | Don't know | 6% | 10% | 7% | 10% | 39% |
| **Car** | Not Comfortable | 22% | 80% | 64% | 31% | 44% |
|  | Comfortable | 76% | 16% | 34% | 65% | 23% |
|  | Don't know | 1% | 4% | 2% | 4% | 33% |
| **Taking back the control of the vehicle** | | | | | | |
| **At any point in time** | Don't know | 1% | 7% | 3% | 8% | 41% |
|  | No | 7% | 52% | 19% | 23% | 24% |
|  | Yes | 92% | 41% | 78% | 70% | 34% |
| **When the vehicle tells that it does not understand the situation** | Don't know | 2% | 7% | 4% | 9% | 44% |
|  | No | 17% | 65% | 35% | 32% | 30% |
|  | Yes | 81% | 28% | 61% | 59% | 26% |
| **In case of emergency** | Don't know | 3% | 7% | 5% | 10% | 44% |
|  | No | 21% | 68% | 43% | 35% | 31% |
|  | Yes | 76% | 25% | 53% | 55% | 24% |
| **To avoid accidents** | Don't know | 6% | 9% | 8% | 12% | 47% |
|  | No | 34% | 74% | 55% | 42% | 34% |
|  | Yes | 61% | 17% | 37% | 46% | 18% |
|  |  |  |  |  |  |  |
|  | | | | | | |
| **Previous experience and knowledge about automated and semi-automated driving assistance systems** | | | | | | |
|  |  | **Profile 1** | **Profile 2** | **Profile 3** | **Profile 4** | **Profile 5** |
| **Experience with automated and semi-automated driving assistance systems** | | | | | | |
| **Auto transmission** | No | 31% | 63% | 41% | 55% | 76% |
|  | Yes | 69% | 37% | 59% | 45% | 24% |
| **Cruise control** | No | 39% | 70% | 46% | 63% | 79% |
|  | Yes | 61% | 30% | 54% | 37% | 21% |
| **Parking assistance** | No | 48% | 80% | 60% | 72% | 87% |
|  | Yes | 52% | 20% | 40% | 28% | 13% |
| **Automated emergency braking** | No | 43% | 75% | 54% | 66% | 84% |
|  | Yes | 57% | 25% | 46% | 34% | 16% |
| **Blind-spot monitoring** | No | 40% | 70% | 49% | 64% | 79% |
|  | Yes | 60% | 30% | 51% | 36% | 21% |
| **Adaptive headlights** | No | 51% | 81% | 63% | 73% | 86% |
|  | Yes | 49% | 19% | 37% | 27% | 14% |
| **Steering assistance** | No | 52% | 82% | 64% | 73% | 88% |
|  | Yes | 48% | 18% | 36% | 27% | 12% |
| **Eletronic stability control** | No | 55% | 80% | 67% | 74% | 85% |
|  | Yes | 45% | 20% | 33% | 26% | 15% |
| **Adaptive cruise control** | No | 44% | 74% | 52% | 68% | 84% |
|  | Yes | 56% | 26% | 48% | 32% | 16% |
| **Lane-keep assistance** | No | 50% | 80% | 61% | 74% | 88% |
|  | Yes | 50% | 20% | 39% | 26% | 12% |
| **Traffic sign recognition** | No | 46% | 74% | 54% | 69% | 82% |
|  | Yes | 54% | 26% | 46% | 31% | 18% |
| **Association of the idea of AVs with different vehicles** | | | | | | |
| **PT picture** | Don't know | 1% | 4% | 1% | 2% | 18% |
|  | No | 22% | 46% | 32% | 30% | 32% |
|  | Yes | 77% | 50% | 66% | 68% | 49% |
| **Truck picture** | Don't know | 1% | 4% | 1% | 3% | 20% |
|  | No | 55% | 74% | 70% | 55% | 55% |
|  | Yes | 44% | 22% | 29% | 42% | 25% |
| **Car picture** | Don't know | 0% | 3% | 1% | 2% | 17% |
|  | No | 14% | 35% | 23% | 18% | 25% |
|  | Yes | 85% | 62% | 77% | 80% | 58% |
|  |  |  |  |  |  |  |
|  |  |  |  |  |  |  |
| **Mobility needs and automated vehicles** | | | | | |  |
|  | **Profile 1** | **Profile 2** | **Profile 3** | **Profile 4** | **Profile 5** |  |
| **Willingness to purchase an AV** | |  |  |  |  |  |
| Affordable | 43% | 1% | 15% | 5% | 3% |  |
| Available | 7% | 0% | 1% | 1% | 0% |  |
| DK | 0% | 2% | 0% | 15% | 29% |  |
| Never | 0% | 92% | 14% | 70% | 58% |  |
| Social Influence | 50% | 5% | 70% | 10% | 10% |  |
| **Willingness to use different automated mobility** | | | | | |  |
|  |  |  |  |  |  |  |
| None | 7% | 75% | 34% | 29% | 49% |  |
| Other | 0% | 0% | 1% | 1% | 3% |  |
| Privately owned AV | 50% | 7% | 26% | 25% | 9% |  |
| AV shared and used as a collective transport service | 12% | 2% | 7% | 9% | 3% |  |
| Hire an AV for individual needs | 17% | 9% | 19% | 19% | 8% |  |
| Shared AVs and used as a ride sharing service | 12% | 3% | 10% | 10% | 3% |  |
| **Willingness to perform activities while the vehicle is driving** | | | | | | |
| **Sleeping** | No | 74% | 92% | 87% | 82% | 91% |
|  | Yes | 26% | 8% | 13% | 18% | 9% |
| **Working** | No | 73% | 96% | 87% | 89% | 95% |
|  | Yes | 27% | 4% | 13% | 11% | 5% |
| **Entertainment** | No | 60% | 90% | 77% | 76% | 87% |
|  | Yes | 40% | 10% | 23% | 24% | 13% |
| **Listening to music or radio** | No | 46% | 82% | 62% | 63% | 79% |
|  | Yes | 54% | 18% | 38% | 37% | 21% |
| **Looking at the scenery** | No | 43% | 73% | 54% | 50% | 71% |
|  | Yes | 57% | 27% | 46% | 50% | 29% |
| **Paying attention to the other vehicles** | No | 59% | 64% | 51% | 70% | 80% |
|  | Yes | 41% | 36% | 49% | 30% | 20% |
|  |  |  |  |  |  |  |
| **Expectations, concerns and policy implications** | | | | | | |
|  |  | **Profile 1** | **Profile 2** | **Profile 3** | **Profile 4** | **Profile 5** |
| **Beliefs regarding the impact of AVs at individual and societal levels,** | | | | | | |
| **Reduce congestion** | No | 60% | 87% | 74% | 74% | 91% |
|  | Yes | 40% | 13% | 26% | 26% | 9% |
| **Increase activities while driving** | No | 61% | 88% | 74% | 76% | 89% |
|  | Yes | 39% | 12% | 26% | 24% | 11% |
| **Reduce the need for professional drivers** | No | 64% | 70% | 63% | 72% | 82% |
|  | Yes | 36% | 30% | 37% | 28% | 18% |
| **Increase accessibility** | No | 67% | 89% | 77% | 77% | 91% |
|  | Yes | 33% | 11% | 23% | 23% | 9% |
| **Reduce stress** | No | 56% | 88% | 76% | 72% | 89% |
|  | Yes | 44% | 12% | 24% | 28% | 11% |
| **Decrease travel time** | No | 70% | 89% | 81% | 77% | 93% |
|  | Yes | 30% | 11% | 19% | 23% | 7% |
| **Reduce emissions** | No | 63% | 84% | 70% | 73% | 89% |
|  | Yes | 37% | 16% | 30% | 27% | 11% |
| **Improve driving comfort** | No | 55% | 88% | 74% | 71% | 89% |
|  | Yes | 45% | 12% | 26% | 29% | 11% |
| **Willingness to share mobility data with different entities** | | | | | | |
| **Road users** | Not Comfortable | 31% | 73% | 61% | 33% | 33% |
|  | Comfortable | 67% | 18% | 35% | 61% | 16% |
|  | Don't know | 2% | 10% | 4% | 7% | 52% |
| **Public authorities** | Not Comfortable | 27% | 69% | 55% | 31% | 31% |
|  | Comfortable | 71% | 22% | 41% | 63% | 18% |
|  | Don't know | 2% | 9% | 3% | 6% | 51% |
| **Private companies** | Not Comfortable | 37% | 75% | 66% | 36% | 33% |
|  | Comfortable | 61% | 15% | 30% | 58% | 15% |
|  | Don't know | 2% | 10% | 4% | 7% | 52% |
| **Importance of different stakeholders for the deployment of Avs** | | | | | | |
| **Private companies** | Don't know | 3% | 17% | 7% | 8% | 51% |
|  | Important | 84% | 54% | 70% | 78% | 40% |
|  | Not Important | 13% | 29% | 23% | 15% | 9% |
| **Public authorities** | Don't know | 3% | 16% | 6% | 7% | 50% |
|  | Important | 87% | 58% | 73% | 79% | 42% |
|  | Not Important | 11% | 26% | 21% | 14% | 8% |
| **European Union** | Don't know | 3% | 17% | 8% | 7% | 51% |
|  | Important | 86% | 56% | 72% | 79% | 42% |
|  | Not Important | 11% | 27% | 20% | 13% | 7% |
| **International organisations** | Don't know | 5% | 20% | 10% | 10% | 56% |
|  | Important | 71% | 47% | 57% | 69% | 33% |
|  | Not Important | 24% | 33% | 32% | 21% | 11% |
|  |  |  |  |  |  |  |
|  |  |  |  |  |  |  |
| **Socio-demographic characteristics and mobility habits** | | | | | | |
|  |  | **Profile 1** | **Profile 2** | **Profile 3** | **Profile 4** | **Profile 5** |
| **Gender** | Man | 58% | 37% | 46% | 47% | 35% |
|  | Woman | 42% | 63% | 54% | 53% | 65% |
| **Internet use at home** | 2 or 3 times a week | 6% | 9% | 7% | 7% | 9% |
|  | 2 or 3 times a month | 0% | 1% | 1% | 1% | 0% |
|  | About once a week | 2% | 3% | 2% | 3% | 1% |
|  | Everyday or almost | 86% | 55% | 79% | 70% | 49% |
|  | Less often | 1% | 2% | 1% | 2% | 3% |
|  | Never | 4% | 25% | 8% | 15% | 28% |
|  | No internet access | 1% | 6% | 1% | 3% | 9% |
| **Age** | 15-24 | 14% | 5% | 10% | 10% | 9% |
|  | 25-39 | 30% | 14% | 23% | 20% | 15% |
|  | 40-54 | 28% | 20% | 29% | 24% | 22% |
|  | 55+ | 28% | 61% | 39% | 46% | 54% |
| **Readiness to pay more to improve mobility** | Don't know | 3% | 5% | 3% | 6% | 17% |
|  | Not ready to pay more | 35% | 61% | 39% | 49% | 62% |
|  | Ready to pay more | 62% | 35% | 57% | 45% | 21% |
| **Main mode of transport** | Car | 59% | 46% | 59% | 44% | 35% |
|  | Don't know | 0% | 0% | 0% | 0% | 0% |
|  | No daily regular mobility | 1% | 4% | 1% | 3% | 6% |
|  | Other | 0% | 0% | 0% | 0% | 0% |
|  | Private bike or scooter | 8% | 7% | 9% | 8% | 4% |
|  | Private motorbike or moped | 2% | 1% | 1% | 1% | 1% |
|  | Ship or boat | 0% | 0% | 0% | 0% | 0% |
|  | Train (non urban) | 2% | 1% | 1% | 1% | 0% |
|  | Urban PT | 15% | 17% | 15% | 21% | 22% |
|  | urban shared bike | 1% | 1% | 1% | 2% | 1% |
|  | Walking | 11% | 21% | 13% | 20% | 29% |
